# Embracing Reablement as an Essential Support Approach for Dementia Care in the 21^st^ Century: A Position Paper

**DOI:** 10.2147/JMDH.S484069

**Published:** 2024-11-25

**Authors:** Silke F Metzelthin, Jette Thuesen, Hanne Tuntland, Magnus Zingmark, Yun-Hee Jeon, Hanne Kaae Kristensen, Lee-Fay Low, Christopher J Poulos, Jackie Pool, Miia Rahja, Erik Rosendahl, Marjolein E de Vugt, Clarissa Giebel, Maud J L Graff, Linda Clare

**Affiliations:** 1 Department of Health Services Research, Maastricht University, Maastricht, the Netherlands; 2 Living Laboratory in Ageing and Long-Term Care, Maastricht, the Netherlands; 3 REHPA, The Danish Knowledge Centre for Rehabilitation and Palliative Care, Nyborg, Denmark; 4 Department of Clinical Research, University of Southern Denmark, Odense, Denmark; 5 Department of Health and Functioning, Western Norway University of Applied Sciences, Bergen, Norway; 6 Department of Occupational Therapy, Institution of Community Medicine and Rehabilitation, Umeå University, Umeå, Sweden; 7 Faculty of Medicine and Health, The University of Sydney, Sydney, NSW, Australia; 8 Centre for Innovative Medical Technology, Department of Clinical Research, University of Southern Denmark, Odense, Denmark; 9 Health Sciences Research Centre UCL University College, Odense, Denmark; 10 Faculty of Medicine & Health, University of New South Wales, Sydney, NSW, Australia; 11 HammondCare, Sydney, NSW, Australia; 12 Dementia PAL, Southampton, UK; 13 Flinders Health and Medical Research Institute, Flinders University, Adelaide, SA, Australia; 14 Department of Community Medicine and Rehabilitation, Umeå University, Umeå, Sweden; 15 Department of Psychiatry and Neuropsychology, Alzheimer Center Limburg, Mental Health and Neuroscience Research Institute, Maastricht University, Maastricht, the Netherlands; 16 Department of Primary Care and Mental Health, University of Liverpool & NIHR Applied Research Collaboration North West Coast, Liverpool, UK; 17 Department of Rehabilitation & Department of IQ health, Radboudumc Alzheimer Center, Radboud University Medical Center Nijmegen, Nijmegen, the Netherlands; 18 University of Exeter Medical School and NIHR Applied Research Collaboration South-West Peninsula, Exeter, UK

**Keywords:** ageing, sustainability, autonomy, capacity-building, functioning, social participation

## Abstract

The World Health Organization (WHO) recognizes the right of individuals with dementia and their family caregivers to access interventions that enhance their participation in society. Reablement is an approach that enables older people to participate in meaningful daily and social activities. Over the past decade, a growing body of evidence has underscored reablement as a promising approach within dementia care, including positive outcomes for people with dementia and their family caregivers, and cost-effectiveness. However, the dissemination of knowledge about and practical implementation of reablement remain slow. This position paper, authored by the ReableDEM research network, aims to address key issues related to implementing reablement in dementia care. To expedite the adoption of reablement within dementia care, we propose five critical areas of focus: 1) *Changing the attitudes and expectations of stakeholders (eg health and social care staff, policy makers, funders)* – encouraging people to think about dementia as a disability from a biopsychosocial perspective; 2) *Disrupting health and social care* - A radical change is needed in the way services are organized so that they are more holistic, personalized and resource-oriented; 3) *Investing in capacity-building and creating a supportive environment* – the workforce needs to be trained and supported to implement reablement in dementia care; 4) *Involving, educating and supporting family caregivers* - services and staff that are equipped to provide reablement will be better able to involve family caregivers and the person’s social network; 5) *Providing robust evidence about reablement in dementia care* by conducting high-quality research with long-term follow-up.

## Introduction

Global ageing has led to a corresponding increase in the number of people with dementia, which makes dementia one of the greatest health and social care challenges of the 21^st^ century. More than 55 million people are currently living with dementia worldwide.^1^ Dementia is typically associated with neurodegenerative diseases characterized by a progression of cognitive and physical decline that causes limitations in performing meaningful daily and social activities.^2^ Functioning is always affected, even in early stages of the disease, and an increased need for support from family caregivers, local community support and professional intermediate- and long-term care is often the consequence.^3^ Despite the decline, people with dementia can have a meaningful life if they receive appropriate psychosocial support.^4^ However, the psychosocial aspects of dementia vary across cultures. In more collectivist societies families bear a significant caregiving burden, often with limited institutional support, leading to isolation and emotional strain. In contrast, Western societies may offer more structured support systems, but stigma and fear of cognitive decline still contribute to emotional stress for both people with dementia and family caregivers.^5^

Currently, the focus in care is on assessing and supporting specific cognitive processes such as memory, perception, language, and executive function without recognizing that cognition can only be facilitated fully within the context of understanding how the person is functioning in everyday life. Therefore, it is important to assess and understand how changes in cognition, along with changes in physical functions, affect functional ability for people with dementia and how personal characteristics and aspects of the physical and social context influence cognitive functioning.^6^ While the search for major pharmacological breakthroughs is critical, medications alone do not solve the challenges faced by people with dementia and their family caregivers. Only a minority of people are eligible for these treatments, and they currently do not offer a cure for the disease.^7^ Therefore, non-pharmacological interventions are urgently needed to adequately support people with dementia. This position paper by the ReableDEM research network argues that reablement should be promoted as a non-pharmacological interdisciplinary approach for people with dementia to improve or maintain their participation in meaningful daily and social activities. Such participation could benefit the quality of life of people with dementia and their family caregivers and reduce the demand for intermediate and long-term care, which will contribute to the sustainability of health and social care systems around the globe. “Reablement” is an approach that enables and empowers people to participate in meaningful daily and social activities by applying person-centered, resource-oriented, and goal-directed methods.^2^,^8^,^9^ Other terms sometimes used to describe this approach include restorative care, function-focused care, and cognitive rehabilitation. The approach is operationalized in a range of intervention programs and service models which demonstrate application of the principles and components of reablement.

## Reablement – a Human Right for People Living with Dementia

In 2017, the World Health Organization (WHO) launched the Global Action Plan for the Public Health Response to Dementia.^10^ According to the WHO, rehabilitative approaches like reablement should be offered to people with dementia and their family caregivers to support them in preserving their autonomy and capability.^2^ This aligns with the message from the International Federation of Ageing. In 2016, they articulated two compelling reasons for reablement in dementia care: sustainability and human rights.^11^ Reablement is sustainable as it can improve the functioning of older people and in the process reduce the need for costly health and social care measures.^11^ The right of people with dementia to have access to comprehensive rehabilitation services, including reablement, is further stressed by the UN Convention on the Rights of Persons with Disabilities.^12^ In 2023, Bickenbach et al argued for human functioning as the third indicator of health, complementing morbidity and mortality, because adding more life to years is even more important than adding years to our lives.^13^ Occupational injustice, a human rights issue, has been documented in several studies highlighting the inequities and barriers individuals face in participating in meaningful daily and social activities.^14^,^15^ Although there is no cure, early recognition and supportive treatment can improve the lives of people with dementia and their family caregivers significantly.^16^

## Reablement - A Promising Health and Social Care Approach in Dementia

**Figure 1:**
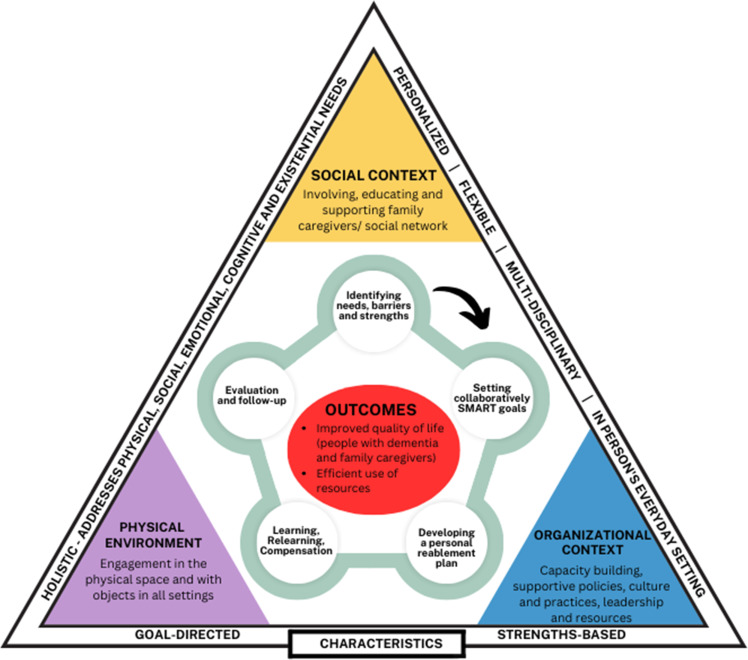
ReableDEM model.

Over the last two decades, reablement has been acknowledged as a promising health and social care approach in many high-income Western countries such as Australia, Denmark, Netherlands, New Zealand, Norway, Sweden, and the United Kingdom.^17^ A conceptual difference can be made between time-limited intervention programs (see Box 1) and service models that are based on the principles of reablement (see Box 2). The core characteristics of both are combined in Figure 1.

**Box 1:** Time-Limited Intervention Programs

These approaches are in line with the international reablement definition.^9^ Examples are “The Care of People with Dementia in Their Environments (COPE)”,^18^,^19^ an interdisciplinary home-based reablement program (I-HARP),^20^,^21^ goal-oriented cognitive rehabilitation (GREAT CR trial),^22^ Community Occupational Therapy in Dementia (COTID)^23–25^ and the Home Care Assistance Program.^26^ All intervention programs focus on people with dementia as well as their family caregivers with the aim of improving their participation in meaningful daily and social activities. They start with identifying needs, barriers and strengths. Subsequently SMART goals are set with people with dementia and their family caregivers (if present) and a personal reablement plan is developed. Various disciplines are involved in the implementation of the plan, eg occupational therapists, physiotherapists, nurses, psychologists and/ or support workers. They support people with dementia and their family caregivers to use new learning or relearning techniques to address the cognitive impairment that is leading to the functional disability. Examples of cognitive rehabilitation learning techniques include action-based learning; mnemonics; expanding rehearsal; prompting and fading. If this approach is unsuitable or unsuccessful, compensatory approaches are taught to address cognitive, functional, and psychosocial difficulties experienced by people with dementia. Examples of compensatory approaches are the use of memos, signage, calendars and information technology. Other interventions may include education (eg about dementia), addressing pain and/ or changes in mood, physical exercises and support for family caregivers. Finally, the achievement of goals is evaluated and if needed follow-up support is arranged. Typically, intervention programs last for 3–4 months and involve 6–12 home visits. The Home Care Assistance program is the most intense intervention program with 3 visits a week. Previous studies have shown positive outcomes regarding participation in activities of daily living. Notably, the I-HARP model enhanced functional independence of people with mild-dementia but did not result in better outcomes for people with moderate to severe dementia. The COPE intervention and Home Care Assistance Program showed an increase in the wellbeing of family caregivers. COTID resulted in decreased caregiver burden, increased quality of life and cost-effectiveness. However, all studies reported that caregivers felt better equipped to cope with the consequences of dementia. In addition, when comparing goal-oriented cognitive rehabilitation to other interventions and usual care, a delay in the transition to residential care by 6 months in the cognitive rehabilitation group relative to usual care was demonstrated while other interventions showed no benefits.^27^

**Box 2:** Service Models Based on the Principles of Reablement

LifeFul,^28^ Function-Focused Care (FFC),^29^,^30^ the SELF-program,^31^,^32^ and SOCAV^33^ are examples of service models based on the principles of reablement. The focus of these programs is on capacity building and creating an environment that facilitates health and social care staff in engaging people with dementia in meaningful daily and social activities. The service models show variation in content, duration (3–12 month), and intensity (1–4 hours/month). They included staff education and training, peer training, assessing policy and practices, implementing new procedures, and developing new policies (eg applying dedicated rostering). Managerial visibility and support are encouraged to reinforce the desired behavior change in staff. Previous research showed that trained staff stimulated people with dementia to actively engage in daily activities more than staff in the control group. In the FFC-CI trial people with dementia also demonstrated a significant improvement in physical activity. The SELF-program was cost-effective regarding improving daily functioning and quality of life. The LifeFul intervention showed improvements in depressive symptoms, functioning, safety, activity engagement, dignity, and overall quality of life for residents. The SOCAV intervention showed increased self-direction of people with dementia and changed self-direction supportive attitudes and behavior of nursing staff.

## Bridging the Evidence-Implementation Gap of Reablement in Dementia Care

Reablement was not originally developed for people with dementia, but in recent years a steady number of approaches for this population group based on principles of reablement have been documented. These show promising results regarding outcomes in people with dementia and their family caregivers, cost-savings, and cost-effectiveness (see Box 1 and Box 2). Nevertheless, reablement in dementia care has spread slowly and has been poorly adopted, despite implementation efforts. This position paper addresses five key issues that might accelerate the adoption of reablement in dementia care: 1) Changing the attitudes and expectations of stakeholders; 2) Disrupting health and social care; 3) Investing in capacity-building and create a supportive environment; 4) Involving, educating and supporting family caregivers; and 5) Providing robust evidence about reablement in dementia care.

### Changing Stakeholders’ Attitudes Towards and Expectations of Dementia

The way in which dementia is understood affects how people with dementia are supported.^34^ A shift from a disease-oriented model of dementia towards a biopsychosocial disability model is a precondition for espousing a reablement approach.^3^ It requires a shift in philosophical and conceptual understanding by individuals and societies: from provision of care to enablement, where people are empowered to direct their own lives.^35^ The understanding of dementia as an interplay between neurological impairment and psychosocial factors, namely, health, individual psychology, and the environment, with particular emphasis on social context, was already promoted three decades ago by Tom Kitwood.^36^ Yet, the potential for reablement is still not fulfilled and should be stressed in public knowledge dissemination. We must acknowledge the abilities of people with dementia from a broad perspective, considering their capacity to maintain their own identity, to participate and contribute to communities, to give and receive support and to participate in shared decision-making.^37^,^38^ Being autonomous, self-reliant, and able to adapt and cope with daily difficulties with or without support of their family caregivers (if available), are central aspects of effective dementia management.^23^,^37^ In addition, care for people with dementia is often centered around basic and instrumental activities of daily living rather than person-centered, meaningful activities at home or in the wider community context. Promoting reciprocal relationships, staying engaged in meaningful social activities and having a sense of belonging in the community can also enhance the well-being of people with dementia and their family caregivers.^23^,^39^

### Disrupting Health and Social Care to Enable Holistic, Personalized and Resource-Oriented Care

Services for people with dementia are often fragmented, hard to access and often depends on the geographical area where people live. Obtaining a diagnosis can be challenging, as healthcare providers are often reluctant to make the diagnosis. Post-diagnostic support and social support services are often lacking and services may be poorly coordinated.^35^ The current health and social care system is built on a disease and deficit model rather than a disability model of dementia.^37^,^38^ Separate programs target different care aspects, for example, function (eg home cleaning and transport), behavior, and caregiver respite, rather than meeting the holistic needs of people with dementia in the context of their family and environment. The current organization and culture of the health and social care system act as barrier.^40^ Therefore, reablement requires disruption rather than adaptation of the current health and social care system.^41^ Structures and practices need to be oriented towards providing accessible integrated care and support, for example, care pathways and funding mechanisms that prioritize rehabilitative approaches like reablement. In addition, it is suggested that governments include quantifiable targets for health and social care providers to refer and deliver this care to people with dementia.^42^ In addition, reablement needs to be implemented at an early stage to support people with dementia and their familiy caregivers.

### Investing in Capacity-Building and Create a Supportive Environment for Reablement Staff

Health and social care staff, who are guided by a disease and deficit-based approach, tend to focus on doing things *for* people with dementia rather than enabling them to do things as independently as possible. Studies have described lack of knowledge, confidence and skills, and reluctance to support enabling approaches in staff working with people with dementia.^43^ Implementing a strengths-based resource-oriented approach-like reablement requires a behavioral change enabling staff to work towards empowering people with dementia to participate in meaningful daily and social activities. To implement such a change, training and coaching is needed. Staff need to understand that people with dementia can benefit from enabling approaches and that the outcomes are worth the investment.^44^ Previous research has shown that interactive and practice-oriented sessions tailored to staff needs create awareness and enhance intrinsic motivation to change behavior.^45^ However, training and coaching alone do not guarantee behavioral change, as reablement often conflicts with regulations that establish standardization, safety and accountability. Shortages in time and resources, standardized protocols, safety regulations, bureaucratic burden or lack of managerial and organizational support can act as barriers. Therefore, an institutional context that facilitates reablement is an important prerequisite for successful implementation, including resources, flexibility, a risk-tolerant approach and broad organizational support and involvement from all stakeholders.^45^ This is in line with the GREAT into Practice’ implementation study in which organizational factors, such as culture, structure, leadership, and resources, were identified as barriers to implementation in health care services and limited the potential for sustainability.^46^ Success in implementing reablement seems to depend on the integration and maintenance of the reablement philosophy within the organization. Management plays a pivotal role in this by establishing a strong network, a shared vision, clear communication, and an innovative climate.^47^

### Involving, Educating and Supporting Family Caregivers of People with Dementia

Family caregivers are a source of ideas and experience that is often not recognized or used.^48^ Involving, educating and supporting family caregivers at an early stage presents a crucial avenue for people with dementia to continue participation in meaningful daily and social activities.^49^ Also previous research has shown that family caregivers are seldom involved in reablement or are insufficiently supported throughout the process, exacerbating caregiver burden.^50^ Often, family caregivers are unaware of the capacities of their relative and focus more on safety concerns than on fostering independence and activities.^38^,^51^ The constant struggle of balancing safety versus autonomy can sometimes lead to involuntary treatment of people with dementia.^52^ This care dilemma underscores the importance of including family caregivers in reablement approaches and ensuring that they have the right information and advice to provide care for their relative.^50^ Family caregivers want more support and recognition of their needs as human being (eg a break from providing care).^50^ They also want to be involved in and participate in decision-making regarding care and to be a contributor to the care of their relative.^53^ Insufficient preparation, lack of information and education, poor communication and collaboration with care professionals, and lack of involvement of family caregivers^54^,^55^ often result in increased anxiety and stress, social isolation, decreased quality of life, financial difficulties, and worsened perceived health of family caregivers.^55–57^ Consequently, family caregivers often become the “invisible second patients”, highlighting the importance of also assessing and addressing the needs of the caregiver alongside those of the care recipient.^49^ In contrary, if a reablement approach is used in which family caregivers are involved in care for people with dementia and are guided in how to effectively support their relative with dementia and at the same time balance this with their own needs, it can lead to a decrease in caregiver burden and can improve the caregiver’s quality of life, mood and health.^23^,^25^,^40^

### Providing Robust Evidence About Reablement in Dementia Care

Another explanation for the slow adoption of reablement in dementia care might be that policy makers are not convinced that the outcomes of reablement in dementia care are worth the investment.^3^ Further high-quality research on reablement in dementia care is needed to drive system change.^11^ Here, we highlight four key areas for future research. First, research must include the perspectives and experiences of people with dementia and their family caregivers. This is essential in ensuring that approaches are designed to meet their needs and to extend understanding and acceptance of reablement as they become empowered to advocate for it. Furthermore, it increases the potential for approaches to be implemented. Second, there is a need to reach consensus on the specific outcomes that are most relevant to the overarching goals of reablement and develop measures that capture those outcomes in a meaningful way. As increasing or maintaining independence is achievable to a limited extent in dementia care, other outcomes, such as self-direction, autonomy, participation, and well-being, might be more relevant. In addition, it is important to evaluate health and well-being of family caregivers. Third, the case for reablement will be strongest if it yields benefits that either extend beyond short-term improvement in key outcomes for people with dementia and family caregivers or reduce the demand for long-term care or both – for example, if it achieves a reduction in need for home care or a delay in nursing home admission. However, intervention studies in this field are rarely designed to evaluate long-term outcomes. Research that recognizes the potential for longer term effects of reablement is needed and funders have a role in encouraging such an opportunity. For example, healthcare insurance data can be used to demonstrate the long-term impact of reablement on people with dementia and family caregivers and enable various aspects of economic evaluation. Fourth, beyond demonstrating benefits in research trials, research must focus on facilitating the implementation of reablement in health and social care by addressing known barriers to improving the accessibility of services and promoting sustainable changes in practice.

## Conclusions

The potential of reablement in dementia care is increasingly recognized, yet its adoption remains slow and inconsistent. To bridge the gap between evidence and implementation, five key areas need to be addressed. First, attitudes towards dementia should be improved by adopting a biopsychosocial disability model. Second, the current health and social care system is fragmented and requires a shift from care provision to enablement. A resource-oriented approach-like reablement is needed, but this requires a disruption of the existing system rather than mere adaptation. Third, capacity-building and organizational factors require attention. Fourth, involving and supporting family caregivers of people with dementia at an early stage is crucial, but they are often insufficiently involved, educated or supported, leading to a higher risk of burden. Lastly, more robust evidence about the effectiveness of reablement is needed to drive system change. By embracing reablement as an essential support approach and addressing these areas, the adoption of reablement in dementia care can be accelerated, ultimately improving the quality of life of people with dementia and their family caregivers. This endeavor requires a collaborative effort from researchers, practitioners, policymakers, and the wider community.
